# Predictors of symptomatic lymphocele after kidney transplantation

**DOI:** 10.1007/s11255-019-02269-0

**Published:** 2019-09-05

**Authors:** Maja Joosten, Frank C. d’Ancona, Wilbert A. van der Meijden, Paul P. Poyck

**Affiliations:** 1 Department of Vascular and Transplant Surgery, Radboudumc, Postbus 9101, 6500 HB Nijmegen, The Netherlands; 2 Department of Urology, Radboudumc, Nijmegen, The Netherlands; 3 Department of Nephrology, Radboudumc, Nijmegen, The Netherlands

**Keywords:** Renal transplantation, Complication, Lymphocele, Risk factors, Predictors

## Abstract

**Purpose:**

The development of a symptomatic lymphocele (SL) is a frequent postoperative surgical complication after kidney transplantation. It may lead to pain and discomfort and cause transplant malfunction or even secondary graft loss. A large cohort of renal recipients was investigated to identify the possible risk factors for SL.

**Methods:**

All renal transplant patients of a single centre were retrospectively analysed for SL between January 2010 and December 2017. The SL group was compared to a control group from the same cohort.

**Results:**

45 out of 1003 transplanted patients developed an SL (incidence 4.5%), on average 50 days after kidney transplantation. SLs developed more in older patients, in those with a PD catheter and in ADKDP as primary diagnosis. Surgical predictors for SLs were venous anastomosis on the external iliac vein, concomitant PD catheter removal, perfusion defects, shorter operating time, splint > 7 days, double J stenting, discharge with drain, low initial drain production and ureteral obstruction. Opening of the peritoneum, re-operation for postoperative bleeding and previous nephrectomy seem protective for developing SL.

**Conclusion:**

We found multiple heterogeneous predictors for SL with a common denominator related to surgical management of the retroperitoneal space, peritoneum and the ureter. Future prospective studies are necessary to evaluate the influence of these variables on the development of SL.

## Introduction

One of the most frequently observed postoperative complications of kidney transplantation is a symptomatic perirenal fluid collection, such as a haematoma, a lymphocele or lymphorrhea. A lymphocele in renal transplant recipients is a pseudo-cystic entity filled with lymph fluid, covered with a hard fibrous capsule, localized around the graft. The incidence of lymphoceles after kidney transplantation varies widely and is reported between 0.03% and 33.9% with a mean incidence of 5.2% [1–8]. Lymphoceles are diagnosed primarily by sonographic imaging and occur from 2 weeks up to 6 months after transplantation with a peak incidence at 6 weeks [9].

Most lymphoceles are asymptomatic and resolve spontaneously or even go unnoticed. Symptomatic lymphoceles (SLs), however, cause pain and discomfort mainly due to the presence of a mass in the lower abdominal quadrant. More severe manifestations are ureteral obstruction, infection or renal vein thrombosis that may lead to secondary graft deterioration or even graft loss. Treatment consists of (repeated) aspiration or drainage of fluid, or more invasively, by open or laparoscopic intraperitoneal marsupialization or fenestration, which in turn may lead to complications as well [10–12]. Despite a variety of treatment options, symptomatic lymphoceles remain difficult to treat and frequently lead to recurrent hospital admissions. This has a large impact on the physical and mental wellbeing of the patient as well as on community or personal financial resources.

Current research has mainly focused on the treatment of choice for lymphoceles. There is no consensus about the risk factors for the development of an SL or how to prevent symptomatic lymphoceles from developing [13–15]. We, therefore, retrospectively investigated a large cohort of renal recipients to identify factors that can predict the development of a symptomatic lymphocele and may be possible risk factors.

We hypothesized that risk factors could be divided into three categories: patient characteristics (e.g. a higher BMI, previous abdominal surgery or a previous kidney transplantation in the same fossa, smoking, atherosclerosis), surgical characteristics (e.g. longer surgery time, more dissection needed) and postoperative characteristics (e.g. need for a splint longer than 7 days, need for a nephrostomy catheter).

## Materials and methods

### Study design and study population

For this retrospective, observational, cohort study, the electronic medical records of all 1003 renal transplant recipients from a single academic centre (Radboudumc, Nijmegen, The Netherlands) were reviewed for the development of a symptomatic lymphocele between January 2010 and December 2017. Symptomatic lymphocele was defined as a symptomatic fluid collection near the graft that required an intervention for the graft or patient. Postoperative or postintervention haematomas, urinomas and abscesses were excluded. Since lymphoceles mostly develop within 6 months after renal transplantation, patients needed to have a follow-up time of at least 6 months.

The control group consisted of 520 patients who were randomly selected from the same cohort and evenly distributed over the cohort period from the beginning of 2014 until the end of 2017 (due to the use of an electronic patient system since 2014). According to regulation, no separate approval of the Medical Center Ethical Review Committee was necessary. Informed consent was waived.

### Kidney transplantation

All operations were performed by a senior surgeon sufficiently trained for kidney transplantation. All recipients were given prophylactic broad-spectrum antibiotics (2 g ceftriaxone iv) 30 min prior to incision.

A retroperitoneal approach via a Gibson incision was routinely used for the arterial and venous anastomoses onto the iliac vessels and to perform a ureteroneocystostomy according to Lich–Gregoir subsequently.

If applicable a PD catheter was surgically removed at the end of the procedure. The ureteroneocystostomy was stented using an external splint for 5–7 days. To shorten the hospital admission time, from 2017 a double J stent was inserted and removed 2–3 weeks later at the outpatient clinic. At the end of the procedure, a wound drain was placed just beside the ureteroneocystostomy.

### Immunosuppressive therapy

All patients received triple immunosuppressive therapy consisting of tacrolimus (trough level 8–12 µg/l), mycophenolic acid and prednisolone. After 6 months, tacrolimus (trough level 5–8 µg/l), prednisolone (0.1 mg/kg/day) and mycophenolic acid dose was decreased and then discontinued, unless the patient had side effects of prednisolone. In these cases, prednisolone was decreased and stopped instead of mycophenolic acid. No induction therapy was given before July 2014, whereas thereafter basiliximab was given as induction immunosuppressant. If patients developed a symptomatic lymphocele, the immunosuppressive therapy was not adjusted.

### Study variables

To assess risk factors for development of a symptomatic lymphocele, study variables were extracted from the electronic patient record. The study variables in this retrospective study were divided into patient characteristics, surgery related and related to the postoperative period. Patient characteristics were gender, age at day of transplantation, weight, body mass index, primary diagnosis for renal failure, cardiovascular history, smoking, diabetes mellitus, hypertension, kidney replacement therapy, type of kidney replacement therapy, duration of peritoneal dialysis previous to transplantation, PD catheter at time of surgery, side of the PD catheter, previous abdominal surgery, previous native nephrectomy (ADKDP patients), radiotherapy of pelvis, previous transplantation in same fossa, total kidney transplants, type of donor (living/deceased), AB0 incompatible donor, and need for anti-T cell therapy in case of rejection.

Surgical characteristics were fossa sidedness, atherosclerosis of the iliac arteries, reconstruction of the iliac arteries, cold ischaemia time, anastomosis time, total surgery time, total blood loss, time on ventilation, whether the peritoneum was opened, removal and side of PD catheter, visible perfusion defects after reperfusion, diuresis and placement of splint or double J. Furthermore, for vascular anatomy of the graft and location of the anastomosis, high was defined as an arterial anastomosis on the common iliac artery or aorta, low arterial anastomosis was defined as an anastomosis on the external iliac artery and high venous anastomosis defined as an anastomosis on the common iliac vein and inferior vena cava and low venous anastomosis was defined as an anastomosis on the external iliac vein.

Postoperative characteristics were defined as postoperative bleeding requiring re-operation, drain production and drain in situ at discharge, re-insertion of a drain, marsupialization or fenestration, splint in situ more than 7 days, transurethral catheter in situ for more than 9 days, ureteral obstruction, placement of nephrostomy catheter, any intervention done and mortality.

### Statistical analysis

Univariate analysis was performed on all individual study variables using an unpaired *t* test to identify variables that were predominantly present in either the SL- or the control group, therefore being possible risk factors for the development of an SL. Data are expressed as mean ± standard deviation for continuous variables and as numbers and percentage for non-metric parameters. A *P* value < 0.05 was considered statistically significant. Statistical analysis was performed using statistical package for social sciences (SPSS) version 25.

## Results

Between January 2010 and December 2017, 1003 patients received a kidney transplantation and 45 patients developed a symptomatic lymphocele. The overall incidence of SLs in this study group was 4.5% (95% CI 3.6–5.8%). Incidence per year varied between 1.0% and 11.0%. SLs were detected after an average of 50 days post-surgery (range 11–244 days); this was on average 43 days after the first ultrasound.

Patients in the SL group had a higher age in comparison with the control group (56 ± 13 vs. 47 ± 17 years, *P *= 0.017), more patients had a PD catheter at the moment of transplantation (22% vs. 18%, *P *= 0.007) and more patients in the SL group were diagnosed with ADPKD (31% vs. 16%, *P *= 0.001).

In the SL group, there were fewer patients with ADPKD that previously had a nephrectomy (0% vs. 10%, *P *= 0.030) and less patients with a PD catheter at the same side as the kidney transplantation (0% vs. 36%, *P *= 0.000). No significant differences were found in all other patient characteristics (Table 1).

**Table 1:** Patient, surgical and postoperative variables

	Control group	SL group	*P* value
Patient variables			
Male	58%; 302/520	60%; 27/45	0.539
Mean age at kidney transplant (years)	47 ± 17	56 ± 13	0.017
Mean weight (kg)	75 ± 19	78 ± 18	0.277
Mean body mass index	25 ± 4.8	26 ± 6.4	0.861
Second or more transplants	19%, 101/520	27%; 12/45	0.340
AB0 incompatible donor	2.9%; 15/520	4,4%; 2/45	0.627
Anti T-cell therapy	8.5%; 44/520	13%; 6/45	0.179
Kidney replacement therapy	76%; 393/520	76%; 34/45	0.924
Peritoneal dialysis	19%; 96/520	22%; 10/45	0.565
Duration of peritoneal dialysis (days)	867 ± 599	862 ± 478	0.979
Haemodialysis	57%; 297/520	53%; 24/45	0.659
Previous kidney transplant in same fossa	4.6%; 24/520	2.2%; 1/45	0.147
Previous abdominal surgery	41%; 211/520	44%; 20/45	0.337
Previous nephrectomy (ADKDP)	10%; 52/520	0%; 0/45	0.030
Radiotherapy of pelvic region	1.5%; 8/520	2.2%; 1/45	0.450
Pre-transplant cardiovascular disease	24%; 127/520	22%; 10/45	0.760
Pre-transplant smoking	17%; 87/520	8.9%; 4/45	0.120
Pre-transplant diabetes mellitus	14%; 72/520	13%; 6/45	0.684
Pre-transplant hypertension	47%; 243/520	42%; 19/45	0.110
Donor type: living	64%; 334/520	62%; 28/45	0.115
Donor type: deceased	36%; 186/520	38%; 17/45	0.499
PD catheter at time of kidney transplant	18%; 96/520	22%; 10/45	0.007
PD catheter at same side as kidney transplant	36%; 35/96	0%; 0/10	0.000
Surgical variables			
Artery anatomy: single	76%; 396/520	69%; 31/45	0.319
Artery anatomy: multiple	24%; 124/520	31%; 14/45	0.319
Sacrifice of renal arteries	7.1%; 37/520	11%; 5/45	0.340
Right fossa	74%; 385/520	76%; 34/45	0.998
Low arterial anastomosis (EIA)	33%; 173/520	36%; 16/45	0.761
High arterial anastomosis (AO and CIA)	67%; 347/520	64%; 29/45	0.761
Low venous anastomosis (EIV)	5.4%; 28/520	11%; 5/45	0.021
High venous anastomosis (IVC and CIV)	95%; 492/520	89%; 40/45	0.021
Mean CIT living donor (min)	127 ± 34	118 ± 39	0.753
Mean CIT deceased donor (min)	771 ± 286	864 ± 260	0.878
Mean anastomosis time (min)	25 ± 10	25 ± 12	0.141
Mean total surgery time (min)	165 ± 41	156 ± 33	0.012
Mean total ventilation time (min)	195 ± 61	188 ± 40	0.626
Peritoneum opened	6.4%; 33/520	0.0%; 0/45	0.000
Removal PD catheter	14%; 73/520	18%; 8/45	0.014
Removed PD catheter at same side as kidney transplant	25%; 18/73	0%; 0/8	0.006
Perfusion defect after anastomosis	7.3%; 38/520	20%; 9/45	0.000
Diuresis on table	91%; 474/520	84%; 38/45	0.510
Atherosclerosis (seen during surgery)	11%; 59/520	6.7%; 3/45	0.610
Reconstruction iliac arteries	1.7%; 9/520	0.0%; 0/45	0.790
Mean total blood loss (ml)	372 ml ± 383 ml	317 ml ± 304 ml	0.610
Double J	11%; 57/520	22%; 10/45	0.000
Postoperative variables			
Drain production (ml)	359 ± 533	147 ± 188	0.011
TUC for more than 9 days	1.5%; 8/520	0.0%; 0/45	0.099
Splint for more than 7 days	0.6%; 3/520	2.2%; 1/45	0.009
Nephrostomy catheter	2.9%; 12/520	2.2%; 1/45	0.906
Drain requirement at discharge	1.3%; 7/520	6.7%; 3/45	0.000
Surgery for postoperative bleeding	2.5%; 13/520	0.0%; 0/45	0.033
Marsupialisation	0.2%; 1/520	20%; 9/45	0.000
Drain re-insertion	1.3%; 7/520	100%; 45/45	0.000
Ureteral obstruction	4.2%; 22/520	18%; 8/45	0.000
Intervention done	6.7%; 35/520	100%; 45/45	0.001
Deceased	1.5%; 8/520	8.9%; 4/45	0.000

Data are expressed as the mean ± standard deviation, percentages or number of patients per cohort

*Ao* aorta, *CIA* common iliac artery, *EIA* external iliac artery, *EIV* external iliac vein, *IVC* inferior vena cava, *CIV* common iliac vein, *CIT* cold ischaemia time, *TUC* trans-urethral catheter

Comparing the SL group to the control group, more SLs were seen in patients that had a low venous anastomosis (11% vs. 5.4%, *P *= 0.021), removal of the PD catheter at the end of the kidney transplantation (18% vs. 14%, *P *= 0.014), perfusion defects of the kidney after completion of the anastomosis and reperfusion (20% vs. 7.3%, *P *= 0.000), and shorter surgery time (156 ± 33 vs. 165 ± 41 min, *P *= 0.012).

In the SL group, there was less removal of the PD catheter located at the same side as the kidney graft (0% vs. 25%, *P *= 0.006) and less opening of the peritoneum (0% vs. 6.4%, *P *= 0.000). No differences were found in all other surgical items (Table 1).

In the SL group, there were more patients that needed a splint for more than seven days (2.2% vs. 0.6%, *P *= 0.009), more usage of double J catheters (22% vs. 11%, *P *= 0.000), more drain requirement at discharge (6.7% vs. 1.3%, *P *= 0.000) and more ureteral obstructions (18% vs. 4.2%, *P *= 0.000).

In contrast to this, there was less production of the postoperative drain in comparison with the control group (147 ± 188 ml vs. 359 ± 533 ml, *P *= 0.011) and less patients that needed re-operation for postoperative bleeding (0% vs. 2.5%, *P *= 0.03). No significant differences were found in the other postoperative parameters (Table 1).

## Discussion

Symptomatic lymphoceles are frequently reported surgical complications after kidney transplantation and may cause devastating harm to the transplant as well as the patient. Since we noticed a peak in the incidence of SLs in our hospital, we investigated our complete cohort over the last 7 years. The overall incidence was 4.5% (range 1.0–11%), which is below the mean 5.2% described in literature [1–8]. The most important finding of this study was the heterogenicity of the predictors. In other words, multiple heterogeneous surgical and non-surgical predictors were related to SL, although a common denominator seems to relate to the management of the retroperitoneal space, peritoneum and the ureter. Furthermore, we found an overall mortality that was sixfold higher in the SL group. Causes of death of the transplant recipients were colon carcinoma (1144 days and 1441 days after kidney transplantation), Epstein–Barr virus-driven post-transplant lymphoproliferative disease (309 days after kidney transplantation), metastatic squamous cell carcinoma of the lung (295 days after kidney transplantation), respiratory insufficiency due to pneumonia (154 and 200 days after kidney transplantation) and metastatic cancer with unknown primary tumour in the control group (665 days and 1354 days after kidney transplantation). In the SL group, the causes were diffuse large B cell cerebral lymphoma (161 days after kidney transplantation), sepsis due to perforation of the colon (764 days after kidney transplantation), infected ascites (2153 days after kidney transplantation) and metastatic cholangiocarcinoma (1606 days after kidney transplantation). Due to the large number of days between kidney transplantation and deceasing (range 154–2153 days), there seems to be no correlation between the two. We are, therefore, unable to explain the higher mortality in the SL group. At the time of kidney transplantation, no patients were known to have a (metastatic) carcinoma. A malignancy was a contra-indication for kidney transplantation. Despite the fact that causes of death did not seem to be directly related to an SL, this finding should encourage further research and awareness in the management of kidney transplant patients. We will further discuss the other predictors for SL that this study has found.

A predictable but not less important patient-related factor in the patient characteristics was age. Older patients receiving a kidney transplant were more at risk of developing an SL. Since more patients are treated at higher age, this effect should be addressed to the patient in light of the need for more invasive treatment and higher mortality risk if an SL occurs. In the literature, age as a predictor for lymphoceles has previously been described for prostatectomy. Older patients are prone to a decreased nutritional status accompanied by hypo-albuminemia and hypo-proteinemia, resulting in impaired tissue healing and prolonged lymphorrhea [16].

High body mass index has been described in the literature to increase the risk of SL [17]. We were not able to establish the same effect in this study.

Autosomal dominant polycystic kidney disease (ADPKD) was the only end-stage renal disease (ESRD) that led to an increase of SLs in this study. Martinez-Ocana et al. also reported this correlation [18]. They suggested that the enlargement of the native kidneys could compress the inferior vena cava, which would result in reduction of the lymphatic flow. This in turn would lead to more lymph fluid around the graft, possibly leading to more lymphoceles [18, 19]. Interestingly, we also found a supporting averse effect since a nephrectomy of a large cystic kidney prior to transplantation reduced the risk of SLs.

One of the surgical parameters that was found to increase the risk of an SL was the use of a low venous anastomosis. This effect has previously been described by Sansalone et al.; they pointed out that dissection around iliac vessels should be done with care [1]. Proper handling of lymph tracts can prevent damage and leakage of lymph fluids. Similarly, Inoue et al. described that an anastomosis onto the external iliac artery was a risk factor for SL [19]. They stated that the lymphatic fluid originates from the recipient’s iliac lymph trunk rather than from the graft kidney. From an anatomical point of view, the lymph tracts around the iliac vessels are part of the lymphatic system of the pelvis. They collect lymph of the genital and urinary organs as well as that of the digestive tract. This system is composed of lymphatic nodes and vessels situated inside the conjunctive tissue, near the organs but especially along the external, internal and common iliac vessels [20, 21]. We, therefore, advise to take great care regarding lymph tracts during dissection along the iliac vessels.

A second predictor for SL was related to the presence of a PD catheter at the time of surgery as well as its subsequent removal, whereas removal of the PD catheter when it was located at the same side of the abdomen as the fossa used for transplantation as well as opening the peritoneum reduced the risk for SL. We are not able to explain why a PD catheter located at the same side as the kidney transplantation would be a protecting factor for the development of SL. PD catheters are often inserted from the midline of the abdomen into cavum Douglasi and subsequently tunnelled to the right or left side of the abdomen. Nevertheless, these findings point to an important role of the peritoneum in the development of SLs. It is well known that peritoneal dialysis changes the composition of the peritoneum. Opening the peritoneum by fenestration is a well-known treatment for symptomatic lymphoceles [22–25]. Intentional fenestration might be an option to prevent SLs when it is safe to remove the PD catheter, at the risk of introducing a possibility of internal herniation of the intestine. The role of the peritoneum in the development of SLs may be a factor to assess in a future prospective study.

Another predictor for SLs was the presence of perfusion defects of the kidney after anastomosis and reperfusion, subsequently followed by partial ischaemia. Ischaemia and reperfusion affect many regulatory systems in the renal tissue and may trigger an immune response, resulting in a distinct inflammatory reaction of the kidney graft [26, 27]. This inflammation in turn leads to the attraction of more lymph fluid, causing accumulation of the lymph fluid around the kidney [28].

Another surgical predictor for SLs was the presence of a splint for more than 7 days and the use of a double J catheter (21 days in situ) when compared to standard 5-day splinting. Our theory is that a plastic or silicone tube leads to irritation of the graft ureter. This irritation may then in turn lead to an inflammatory reaction resulting in an accumulation of lymph fluid and hence more symptomatic lymphoceles [29–31]. Irrespective of the type of tubing, splint or double J catheter, the duration seems to be the key factor. This finding is supported by the observation that the double J catheter was introduced in 2017; the same year a significant increase in SL was noted (14.7% double J vs. 7.0% splint). We searched the literature to find a relation between type of stenting and its duration versus SL, but no reports were found. The wide variety in the reported incidences for SL might be related to the postoperative management of the graft ureter and ureteroneocystostomy.

This study showed that drain production was inversely related to the development of SLs. Apparently, less drain production leads to more SLs. Our hypothesis is that improper drainage of the retroperitoneal space directly after the kidney transplantation not only leads to accumulation of the non-drained blood and lymph fluid, but also facilitates more lymph fluid production as reaction to it and thus leads to an SL. This idea is supported by the finding that less SLs were seen in patients requiring a second operation for a postoperative bleeding, wherein blood is removed from the retroperitoneal space, fluid leakages are managed and a drain is properly (re-) replaced. We also investigated the effect of a thin versus a thick drain, but no significantly differences in the outcome of SLs were found. We advocate proper drain placement, advise to keep the drain open and to not remove the drain too quickly to prevent possible formation of an SL.

The slight increase in incidence of SLs over time might be attributed by the use of a double J as described earlier. Since the use of a double J was effectuated from 2017, this partly explains the increase. We, therefore, investigated other parameters that changed in time. A smaller drain diameter was used by May 2014, but no significant difference in SL formation was seen between the period prior and after the introduction. The same holds for the introduction of basiliximab as induction therapy starting July 2014. The trend towards more SLs is, therefore, unaccounted for.

## Limitations

This is a retrospective study, which constitutes an important limitation. However, we obtained complete and detailed information from our database covering multiple years. Another limitation is the fact that these results are based on a single-centre study. Further research must show whether this applies to other settings as well.

Furthermore, since our group of patients that developed a symptomatic lymphocele was relatively small, i.e. out of 1003 patients only 45 did develop a symptomatic lymphocele, we were unable to do an extensive multivariate analysis.

## Conclusion

This study showed that symptomatic lymphoceles develop in one out of 25 patients (4.5%) receiving a kidney transplant and cause significant morbidity and mortality.

We found multiple surgical and non-surgical predictors for the development of a symptomatic lymphocele. The common denominators are the surgical management of the retroperitoneal space and peritoneum, management of the PD catheter at the moment of transplantation, wound drain production and the duration of stenting of the ureteroneocystostomy.

A prospective study is needed to confirm the role of these risk factors in the development of symptomatic lymphoceles. This study may lead to a better awareness and hopefully prevention of development of symptomatic lymphoceles in the future.

## Abbreviations

ADPKD: Autosomal dominant polycystic kidney disease
Ao: Aorta
BMI: Body mass index
CI: Confidence interval
CIA: Common iliac artery
CIT: Cold ischaemia time
CIV: Common iliac vein
EIA: External iliac artery
EIV: External iliac vein
ESRD: End-stage renal disease
IVC: Inferior vena cava
PD: Peritoneal dialysis
SL: Symptomatic lymphocele
TUC: Trans-urethral catheter
